# Utility of a near real-time emergency department syndromic surveillance system to track injuries in New York City

**DOI:** 10.1186/s40621-015-0044-5

**Published:** 2015-06-01

**Authors:** Kacie Seil, Jennifer Marcum, Ramona Lall, Catherine Stayton

**Affiliations:** 1 Bureau of Environmental Disease and Injury Prevention, NYC Department of Health and Mental Hygiene, New York, NY USA; 2 1100 West 49th Street, Room 704.11, Austin, TX 78714 USA; 3 Bureau of Communicable Diseases, NYC Department of Health and Mental Hygiene, New York, NY USA

**Keywords:** Public health surveillance, Epidemiology, Wounds and injuries, Emergency service, hospital

## Abstract

**Background:**

The New York City emergency department (ED) syndromic surveillance (SS) system provides near real-time data on the majority of ED visits. The utility of ED SS for injury surveillance has not been thoroughly evaluated. We created injury syndromes based on ED chief complaint information and evaluated their utility compared to administrative billing data.

**Methods:**

Six injury syndromes were developed: traffic-related injuries to pedal cyclists, pedestrians, and motor vehicle occupants; fall-related injuries; firearm-related injuries; and assault-related stabbings. Daily injury counts were compared for ED SS and the administrative billing data for years 2008–2010. We examined characteristics of injury trends and patterns between the two systems, calculating descriptive statistics for temporal patterns and Pearson correlation coefficients (*r*) for temporal trends. We also calculated proportions of demographic and geospatial patterns for both systems.

**Results:**

Although daily volume of the injuries varied between the two systems, the temporal patterns were similar (all *r* values for daily volume exceeded 0.65). Comparisons of injuries by time of day, day of week, and quarter of year demonstrated high agreement between the two systems—the majority had an absolute percentage point difference of 2.0 or less. Distributions of injury by sex and age group also aligned well. Distribution of injury by neighborhood of residence showed mixed results—some neighborhood comparisons showed a high level of agreement between systems, while others were less successful.

**Conclusions:**

As evidenced by the strong positive correlation coefficients and the small absolute percentage point differences in our comparisons, we conclude that ED SS captures temporal trends and patterns of injury-related ED visits effectively. The system could be used to identify changes in injury patterns, allowing for situational awareness during emergencies, timely response, and public messaging.

**Electronic supplementary material:**

The online version of this article (doi:10.1186/s40621-015-0044-5) contains supplementary material, which is available to authorized users.

## Background

Emergency department (ED) syndromic surveillance (SS), defined as the categorization and monitoring of patient symptoms or diagnosis codes, has become an important resource for public health (Johansen et al. 2011; Buehler et al. 2009; Conway et al. 2013; Paterson and Durrheim 2013; Rosenkotter et al. 2014; Samoff et al. 2012; Mathes et al. 2011; Josseran et al. 2010). SS was created for the early detection of potential bioterrorism agents and communicable diseases, and has been adapted to monitor non-communicable and chronic disease trends (Paterson and Durrheim 2013). Patient chief complaint, a short phrase entered by an admission clerk or triage nurse that details the reason for the patient’s visit, is used to build syndromes that capture a variety of health events (Conway et al. 2013). One salient advantage of ED SS is that it provides near real-time data that is not dependent on laboratory-confirmed diagnoses, which can take several days. Alternatively, administrative discharge data collected from billing information are based on confirmed clinical diagnoses and are often not available until a year or more after the visit. Both data sources provide secondary data collected for purposes not specific to public health surveillance.

The utility of ED SS for injury surveillance has not been thoroughly evaluated. Published works on eye injuries (CDC 2013; Elliot et al. 2010), carbon monoxide poisonings (Buehler et al. 2009; Baer et al. 2011; Chen et al. 2013), drug overdoses (Modarai et al. 2013), animal bites (Bregman and Slavinski 2012; Rhea et al. 2012), hyperthermia (Josseran et al. 2010; Josseran et al. 2006; Josseran et al. 2009; Perry et al. 2011; Schaffer et al. 2012; CDC 2006), hypothermia (Hope et al. 2008), and trauma (Hope et al. 2008) typically do not include an evaluation of efficacy. Use of ED SS for tracking traffic-related injuries, falls, assault-related stabbings, and firearm-related injuries has yet to be evaluated.

New York City’s (NYC) ED SS was developed in 2001, making it one of the first systems of its kind established in the USA (Lederberg et al. 2003). The NYC ED SS currently receives data 365 days a year and covers an estimated 98 % of NYC ED visits. The system is primarily used to identify aberrations and patterns in ED visits for acute illness syndromes, such as diarrhea, vomit, influenza-like illness, asthma, and respiratory symptoms.

SS systems are no longer used exclusively for outbreak detection and early detection of emerging diseases (Paterson and Durrheim 2013; Rosenkotter et al. 2014; Buehler et al. 2009). The NYC ED SS has also been used during weather-related events as a means of monitoring health impacts and providing situational awareness in near real-time (e.g., NYC blackout in 2003, Hurricane Sandy in 2012); these expanding uses of SS are occurring in other cities, states, and countries as well (Paterson and Durrheim 2013; Rosenkotter et al. 2014; Buehler et al. 2009). SS systems are increasingly being used to monitor population health during and after events like heat waves and mass gatherings to inform response and risk messaging to the public (Paterson and Durrheim 2013; Rosenkotter et al. 2014; Buehler et al. 2009).

While ED SS is timely, a consequence is a lack of syndrome specificity. ED SS is not a system that is relied upon for accurate case counts. Some ED-based syndromic surveillance systems do include complete diagnosis information (e.g., NC DETECT (Rappold et al. 2011; Hakenewerth et al. 2009)). The NYC Department of Health and Mental Hygiene (DOHMH) is currently helping hospitals meet meaningful use requirements for ED SS so that they might start sending ED data from certified clinical systems; DOHMH is also requiring hospitals to provide more complete information for key fields (e.g., discharge diagnosis, disposition). Challenges in relying on chief complaint information for syndrome definitions often arise. Chief complaint is based on presenting symptoms, while diagnosis is based on clinical treatment. Text entered in the chief complaint field does not always match the final clinical diagnosis (i.e., the chief complaint may not be reflective of the ailment that is treated in the ED). The chief complaint field is not uniform—it contains a mix of lay language, technical language, misspellings, and abbreviations. Additionally, the NYC ED SS does not include external cause of injury codes (E-codes), which identify the mechanism and intent of injury. For instance, an injury might be a fracture, while the external cause of the injury is an unintentional fall.

The purpose of this project was to create injury syndromes based on NYC DOHMH injury prevention priorities and evaluate the utility of the syndromes compared to administrative ED billing data to inform potential surveillance uses of the syndromes.

## Methods

### Creating injury syndromes

Using NYC ED SS data, six injury syndromes were developed for the following: traffic-related injuries to pedal cyclists, pedestrians, and motor vehicle occupants; fall-related injuries; firearm-related injuries; and assault-related stabbings. We identified phrases and key words used in the chief complaint to classify visits into injury syndromes. Syndrome terms included standard injury mechanism terms (e.g., “assault”) and lay language used to describe the injury (e.g., “stumble”). Cases were reviewed, and syndromes were refined to include common misspellings (e.g., “bicyle”) and abbreviations (e.g., “gsw” for gunshot wounds). Specific terms were also used to exclude conditions clearly not related to the injury of interest (e.g., “bloodshot”). Syndromes were iteratively reviewed and refined until additional exclusion criteria were determined to no longer make substantial impact on the number of cases identified. The syndrome definitions and SAS code used are found in Additional file 1.

### Evaluating injury syndromes

#### Data sources

As of 2015, the NYC ED SS receives information from 51 out of the 53 NYC hospitals, which accounts for 98 % of the daily volume. There are approximately 11,000 ED visits per day in NYC. The NYC ED SS has been operating since 2001 and is a “home-built” system with individual hospitals submitting text-based files or HL7 messages daily. Hospitals submit a 7-day file daily to allow for the backfill of missing information. Injury-related ED visits during 2008–2010 were identified in ED SS with the Perl regular expression (PRX) function in SAS version 9.2 (SAS Institute, Cary, NC), which searches the chief complaint text field for patterns of characters to include or exclude.

The New York State Department of Health Statewide Planning and Research Cooperative System (SPARCS) collects information on hospital discharges, including inpatient stays and outpatient visits (NYS Department of Health 2014). All relevant ED visits were included from the SPARCS outpatient dataset, as well as those in the inpatient dataset who were admitted from that hospital’s ED. Injury-related ED visits were identified using E-codes in the International Classification of Diseases, Ninth Revision, Clinical Modification (ICD-9-CM) system. E-codes used to identify traffic-related injuries to pedal cyclists, pedestrians, and motor vehicle occupants and injuries resulting from falls, firearms, and assault-related stabbings are detailed in Additional file 2.

SPARCS data rely on diagnosis codes provided for billing purposes, compared to ED SS data, which rely on chief complaint. For this reason, SPARCS is the gold standard for enumerating and describing ED visits and hospitalizations. Since SPARCS data are typically available 1 to 2 years after the date of service, our evaluation used the most recently available data (2008–2010). The number of hospitals compared in this evaluation was 47.

#### Evaluation

We examined injury syndrome volume and patterns with the goal of determining whether ED SS can be used to enumerate incident cases and identify aberrations from expected trends and patterns by time, person, and place characteristics compared to SPARCS. First, we compared the daily counts of each external cause of injury for the years 2008–2010 in both the ED SS and SPARCS datasets. To compare injury volume, we calculated the 3-year total and daily mean, median, and standard deviation of ED visits in both the ED SS and SPARCS datasets for the study period. We then calculated Pearson correlation coefficients (*r*) to assess the extent to which the temporal trends were related in the two systems.

Second, we evaluated the temporal patterns for the injury syndromes, calculating the distribution of time of day, day of week, and quarter of year for ED SS and SPARCS. We also calculated the absolute percentage point difference to demonstrate the degree to which the distributions aligned.

Third, we evaluated demographic and geospatial patterns for the injury syndromes, calculating the distribution of sex, age group, and neighborhood of residence for ED SS and SPARCS. We calculated the absolute percentage point difference for the sex, age group, and neighborhood of residence (based on patient’s given zip code of residence) distributions to demonstrate the degree to which they aligned.

## Results and discussion

We first examined the extent to which data were missing for all variables of interest. Although not all variables in ED SS exhibit high levels of completeness, key variables, such as date and time of visit, sex, age, chief complaint, and zip code of residence, have high percentages of completeness. However, one major issue with the chief complaint field is a high percentage of missing data for Staten Island hospitals; two of the three Staten Island hospitals currently provide chief complaint data for only 26% of ED visits. In this evaluation, both systems demonstrated very little missing data (<1 %) overall for all variables of interest. The exception was patient’s zip code of residence, which was used to determine neighborhood of residence. The majority of the “missing” data were zip codes outside of New York City, meaning that the patient could not be assigned a neighborhood of residence. The amount of missing data for that variable was estimated to be ≤3.2 % for both data systems.

All Pearson correlation coefficient (*r*) values for daily volume exceeded 0.65. Values ranged from 0.67 for pedestrian hit by motor vehicle to 0.87 for fall-related injury (see Table 1). In general, ED SS identified fewer injury cases than SPARCS, with the exception of motor vehicle occupant injuries. The daily volume of the injuries varied between the two systems, although firearm and assault-related stabbing injuries had similar volumes in both ED SS and SPARCS, suggesting that these are well documented in the chief complaint. In contrast, SS identified less than 40 % of the total SPARCS volume for cyclist injuries (*N* = 7841 vs. *N* = 20,790, respectively). The difference in volume did not dictate strength of correlation, however.

**Table 1:** Daily counts of injury-related ED visits, ED SS vs. SPARCS, NYC, 2008–2010

	ED SS daily counts, 2008–2010			SPARCS daily counts, 2008–2010			Correlation coefficient (*r*)
	3-year	Daily	Daily	3-year	Daily	Daily	
Injury type	Total *N*	Mean (±SD)	Median	Total *N*	Mean (±SD)	Median	
Traffic-related injury to pedal cyclist	7841	7.2 (±5.3)	6	20,790	19.0 (±13.2)	16	0.85
Traffic-related injury to pedestrian	17,702	16.2 (±5.8)	16	28,815	26.3 (±8.3)	25	0.67
Traffic-related injury to motor vehicle occupant	126,117	115.1 (±24.3)	114	103,767	94.7 (±19.7)	93	0.82
Fall-related injury	312,234	284.9 (±44.1)	284	526,817	480.7 (±67.3)	478	0.87
Firearm-related injury	4596	4.2 (±3.0)	4	5208	4.8 (±3.2)	4	0.82
Assault-related stabbing injury	13,186	12.0 (±5.7)	11	14,618	13.3 (±6.7)	12	0.76

Comparisons of injuries by time of day, day of week, and quarter of year had high agreement between ED SS and SPARCS—the majority of comparisons had an absolute percentage point difference of 2.0 or less. According to both ED SS and SPARCS, 40.1 % firearm-related ED visits occurred between the hours of midnight and 5:59 AM (see Table 2). About 20.5 % of stabbing-related ED visits in ED SS occurred on Sundays, compared to 22.2 % in SPARCS (see Table 3). Both ED SS and SPARCS found that the quarter of the year with the most cyclist-related injuries was July–September, with proportions of 42.4 and 43.0 %, respectively (see Additional file 3).

**Table 2:** Distribution of injury-related ED visits by time of day, ED SS vs. SPARCS, NYC, 2008–2010

	3-year totals, 2008–2010^a^			
Injury type	Time of day	ED SS proportion	SPARCS proportion	Absolute % point difference
Traffic-related injury to pedal cyclist	Midnight–5:59 AM	8.3	9.3	1.0
	6 AM–11:59 AM	16.6	16.2	0.4
	Noon–5:59 PM	36.0	34.4	1.6
	6 PM–11:59 PM	39.0	40.2	1.2
Traffic-related injury to pedestrian	Midnight–5:59 AM	8.4	10.4	2.0
	6 AM–11:59 AM	20.4	20.7	0.3
	Noon–5:59 PM	33.7	33.9	0.2
	6 PM–11:59 PM	37.1	35.0	2.1
Traffic-related injury to motor vehicle occupant	Midnight–5:59 AM	13.6	12.9	0.7
	6 AM–11:59 AM	20.8	21.5	0.7
	Noon–5:59 PM	32.9	34.3	1.4
	6 PM–11:59 PM	32.1	31.3	0.8
Fall-related injury	Midnight–5:59 AM	9.7	9.6	0.1
	6 AM–11:59 AM	22.6	21.2	1.4
	Noon–5:59 PM	36.8	37.3	0.5
	6 PM–11:59 PM	30.4	31.9	1.5
Firearm-related injury	Midnight–5:59 AM	40.1	40.1	0.0
	6 AM–11:59 AM	9.4	14.6	5.2
	Noon–5:59 PM	17.2	15.4	1.8
	6 PM–11:59 PM	32.8	30.0	2.8
Assault-related stabbing injury	Midnight–5:59 AM	36.3	36.4	0.1
	6 AM–11:59 AM	14.6	16.9	2.3
	Noon–5:59 PM	19.2	18.9	0.3
	6PM–11:59 PM	29.1	27.8	1.3

^a^Column percentages may not add up to 100 % due to rounding and/or missing data

**Table 3:** Distribution of injury-related ED visits by day of week, ED SS vs. SPARCS, NYC, 2008–2010

	3-year totals, 2008–2010^a^			
Injury type	Day of week	ED SS proportion	SPARCS proportion	Absolute % point difference
Traffic-related injury to pedal cyclist	Sunday	16.4	15.9	0.5
	Monday	14.2	14.8	0.6
	Tuesday	13.1	13.8	0.7
	Wednesday	13.7	13.2	0.5
	Thursday	13.6	13.2	0.4
	Friday	13.6	13.6	0.0
	Saturday	15.4	15.5	0.1
Traffic-related injury to pedestrian	Sunday	11.1	11.4	0.3
	Monday	14.2	14.1	0.1
	Tuesday	15.2	14.8	0.4
	Wednesday	15.3	15.5	0.2
	Thursday	15.4	15.6	0.2
	Friday	15.7	16.0	0.3
	Saturday	13.0	12.8	0.2
Traffic-related injury to motor vehicle occupant	Sunday	15.0	14.8	0.2
	Monday	14.2	14.2	0.0
	Tuesday	14.0	14.2	0.2
	Wednesday	13.7	13.8	0.1
	Thursday	13.7	13.6	0.1
	Friday	14.6	14.5	0.1
	Saturday	14.9	15.0	0.1
Fall-related injury	Sunday	13.1	13.4	0.3
	Monday	15.2	15.3	0.1
	Tuesday	14.7	14.7	0.0
	Wednesday	14.6	14.6	0.0
	Thursday	14.5	14.3	0.2
	Friday	14.4	14.2	0.2
	Saturday	13.4	13.5	0.1
Firearm-related injury	Sunday	21.7	21.7	0.0
	Monday	14.1	13.8	0.3
	Tuesday	11.8	12.2	0.4
	Wednesday	10.4	10.0	0.4
	Thursday	10.6	10.6	0.0
	Friday	12.2	12.0	0.2
	Saturday	19.2	19.8	0.6
Assault-related stabbing injury	Sunday	20.5	22.2	1.7
	Monday	12.6	11.8	0.8
	Tuesday	11.5	11.6	0.1
	Wednesday	10.9	10.3	0.6
	Thursday	12.1	11.1	1.0
	Friday	12.9	13.0	0.1
	Saturday	19.6	20.1	0.5

^a^Column percentages may not add up to 100 % due to rounding and/or missing data

Tables 4 and 5 display results comparing sex and age group distributions in ED SS and SPARCS for each of the injury types. The maximum difference across all comparisons between ED SS and SPARCS was cyclist injuries by sex. There was a 7.2 absolute percentage point difference between ED SS and SPARCS in the percent of cyclist injuries among males. However, both ED SS and SPARCS show similar patterns, with males comprising the vast majority of cyclist injuries across both systems (72.5 vs. 79.7 %, respectively). For stabbing injuries, the distributions of ED visits by sex were even closer between ED SS and SPARCS, 84.5 % male in ED SS and 84.4 % male in SPARCS (see Table 4). When the proportions of injury were examined by age group, all syndromes showed a high level of agreement across surveillance systems. For example, ED SS found that older adults (ages 65 and older) constituted 27.9 % of fall-related ED visits compared to 23.1 % of fall-related ED visits in SPARCS. Older adults constitute a much smaller proportion of firearm injuries in both ED SS and SPARCS, 0.6 and 0.8 %, respectively (see Table 5).

**Table 4:** Distribution of injury-related ED visits by sex, ED SS vs. SPARCS, NYC, 2008–2010

	3-year totals, 2008–2010			
Injury type	Sex	ED SS proportion	SPARCS proportion	Absolute % point difference
Traffic-related injury to pedal cyclist	Male	72.5	79.7	7.2
Traffic-related injury to pedestrian	Male	57.0	52.6	4.4
Traffic-related injury to motor vehicle occupant	Male	51.7	50.0	1.7
Fall-related injury	Male	44.1	47.2	3.1
Firearm-related injury	Male	91.5	91.1	0.4
Assault-related stabbing injury	Male	84.5	84.4	0.1

**Table 5:** Distribution of injury-related ED visits by age group, ED SS vs. SPARCS, NYC, 2008–2010

	3-year totals, 2008–2010^a^			
Injury type	Age group	ED SS proportion	SPARCS proportion	Absolute percentage point difference
Traffic-related injury to pedal cyclist	0–17	28.5	33.5	5.0
	18–24	17.6	16.2	1.4
	25–44	34.7	33.2	1.5
	45–64	16.1	14.8	1.3
	65+	3.1	2.3	0.8
Traffic-related injury to pedestrian	0–17	22.6	22.4	0.2
	18–24	14.8	13.6	1.2
	25–44	29.9	28.9	1.0
	45–64	22.3	24.3	2.0
	65+	10.4	10.8	0.4
Traffic-related injury to motor vehicle occupant	0–17	15.0	10.3	4.7
	18–24	17.9	18.1	0.2
	25–44	41.0	43.0	2.0
	45–64	21.5	23.1	1.6
	65+	4.7	5.5	0.8
Fall-related injury	0–17	26.9	30.6	3.7
	18–24	5.7	6.2	0.5
	25–44	17.8	18.8	1.0
	45–64	21.6	21.3	0.3
	65+	27.9	23.1	4.8
Firearm-related injury	0–17	12.4	13.4	1.0
	18–24	39.9	39.0	0.9
	25–44	41.2	40.5	0.7
	45–64	5.8	6.3	0.5
	65+	0.6	0.8	0.2
Assault-related stabbing injury	0–17	11.4	12.4	1.0
	18–24	32.1	33.7	1.6
	25–44	43.1	42.6	0.5
	45–64	12.3	10.7	1.6
	65+	1.1	0.7	0.4

^a^Column percentages may not add up to 100 % due to rounding and/or missing data

A comparison of the geographic distributions of the injury syndromes for ED SS and SPARCS demonstrated mixed results. Across neighborhoods, traffic-related injury to pedal cyclist had the highest mean absolute percentage point difference (0.7 %); in contrast, the firearm-related injury comparison had the lowest mean absolute percentage point difference across neighborhoods (0.4 %). Comparing neighborhood of residence was very successful in some cases—for instance, ED SS found that 2.7 % of ED visits for pedestrian hit by motor vehicle were among Washington Heights-Inwood, Manhattan residents, compared to 2.8 % in SPARCS (see Additional file 4). For others, the comparison did not show a high level of agreement—for example, ED SS found that 0.8 % of ED visits for motor vehicle occupant injuries were among South Beach-Tottenville, Staten Island residents, compared to 4.8 % in SPARCS. Two of the three hospitals in Staten Island have a majority of the chief complaint data missing, which would help explain the observed discrepancy in results. Therefore, results for Staten Island should be interpreted with caution.

## Conclusions

This study presents findings of a formal evaluation of an ED SS system for the tracking of specific external causes of injury. Since NYC injury surveillance has traditionally relied upon SPARCS data for understanding counts, trends, and patterns, this evaluation was focused on understanding how ED SS compares to a known and familiar data source: SPARCS. We found strong positive correlation coefficients and small absolute percentage point differences in our ED SS and SPARCS comparisons, leading us to conclude that ED SS captures overall trends and patterns of injury-related ED visits effectively.

This evaluation had a number of limitations. ED SS and SPARCS are two secondary sources of data collected for different purposes. ED SS and SPARCS collect data about the ED visit at two different time points of the visit. ED SS relies on chief complaint text that is collected when the patient enters an ED and first encounters a hospital registrar or a triage nurse. The NYC ED SS system does not currently have complete diagnosis or discharge disposition information to provide complete details about the visit outcome, limiting us to the use of chief complaint data only for syndromic surveillance. The chief complaint text that is entered into ED SS can be verbatim what the patient says or be a summary of the reason for the visit that is free-text or from a drop-down menu. SPARCS, in contrast, provides standardized disease codes collected for billing information and is based on the final diagnosis at discharge provided by the ED doctor after the patient has been examined or treated. Although this makes the comparison of trends between the two data sources a bit more challenging, this evaluation provides us with some guidance and understanding of the utility of ED SS for injury surveillance. Another limitation is that we did not attempt to match patients between ED SS and SPARCS to fully evaluate information available in each of the systems. This may provide better understanding of the discrepancies between the two data systems.

ED SS relies on the querying of the chief complaint text field for key words that reflect injury as the cause for the ED visit and is, therefore, reliant on the quality and completeness of chief complaint field. Since the ED SS system focuses mainly on chief complaint, we neglect to capture some injuries. We hypothesize that some injury cases are described by injury outcome (e.g., laceration, fracture) rather than external cause (e.g., fall) in the chief complaint field. Fall-related injuries can lead to a wide range of injuries, while firearm and stabbing-related injuries are more specific; in those instances, the external cause is more likely to be captured by the chief complaint. Syndrome development and validation is a challenging and iterative process of including and excluding words to ensure that visits being captured truly reflect the syndrome of interest. While it is easy to recognize and exclude words that might falsely identify irrelevant ED visits, it is much harder to determine true injury-related visits we are missing. It is also important to note that syndrome definitions might need to be updated over time to include new medical terminology or words/abbreviations used to describe visits of interest. As syndromic data are being provided in near real-time and currently data are only backfilled for up to the past 7 days, data quality issues must be carefully considered when interpreting results.

Given that injury syndromes in ED SS are based on the chief complaint text field, we do not recommend using this data to determine case counts. The discrepancies in counts for injuries in ED SS versus SPARCS attest to this. While case counts were not reliable, trends and patterns of injury visits were reliably captured by ED SS. The positive *r* values indicate that when the number of ED visits increases in one system, it also increases in the other system, providing confidence in the utility of syndromic surveillance for monitoring injury trends in near real-time. Further, the injury syndromes are able to accurately describe patterns by sex and age group, though geographic patterns are not always as reliable when compared to SPARCS. Differences we see by neighborhood could possibly be due to data quality issues or inconsistencies in hospitals reporting in those neighborhoods and need to be investigated further.

Despite shortcomings, ED SS is a valuable source of timely information; it has a lag of a few hours compared to SPARCS, which has a lag of more than 1 year. The ED SS is not meant to replace traditional surveillance systems, such as SPARCS, but is instead a tool for providing current and timely information to be considered alongside traditional systems and other sources of information during routine daily surveillance or during emergencies (Paterson and Durrheim 2013; Buehler et al. 2009). ED SS provides a means for comparing current trend patterns to what we have seen in the recent past (e.g., past 8 weeks) and providing insights on how syndromic trends might be changing.

ED SS is a tool that can alert city officials to changes in injury patterns and allow for timely response and messaging. For example, during cold weather months, ED SS has been used to track falls. Both ED SS and SPARCS demonstrate large increases in fall-related ED visits following events of freezing rain and snow. Findings could be utilized to inform messaging to the public about falls prevention during inclement weather. On a more routine basis, ED SS can be used to track cyclist injuries daily to monitor injuries potentially related to increased bicycle use and the new Citi Bike Share program in NYC. Given that the firearm and stabbing syndromes appeared to be captured reliably by chief complaint, another alternate use of these syndromes would be for informing community-based youth violence prevention programming of potential cases in near real-time.

The NYC ED SS is a powerful tool that allows public health professionals to monitor the health of the population in near real-time. It can reliably be used to track injuries, as evidenced by this evaluation. Future research projects may include matching cases between ED SS and SPARCS, calculating diagnostic measures such as sensitivity and specificity, conducting random chart reviews, and assessing the possibility for surveillance related to electronic health records in the primary care setting. We would recommend concerted efforts aimed at improving ED SS data quality and completeness, especially more complete reporting of diagnosis codes. We would also support the inclusion of E-codes in the ED SS system.

## Additional files

Additional file 1:
**Injury syndrome inclusion/exclusion criteria.**
Additional file 2:
**ICD-9-CM E-codes for injury types.**
Additional file 3:
**Distribution of injury-related ED visits by quarter of year, ED SS vs. SPARCS, NYC, 2008-2010.**
Additional file 4:
**Distribution of injury-related ED visits by neighborhood of residence, ED SS vs. SPARCS, NYC, 2008-2010.**

## Abbreviations

DOHMH: Department of Health and Mental Hygiene
E-code: external cause of injury code
ED: Emergency department
ICD-9-CM: International Classification of Diseases, Ninth Revision, Clinical Modification
NYC: New York City
PRX: Perl regular expression
SPARCS: Statewide Planning and Research Cooperative System
SS: syndromic surveillance
