# Supplementary material for: Utility of a near real-time emergency department syndromic surveillance system to track injuries in New York City
Source: Inj Epidemiol. 2015 Jun 1;2(1):11. doi: 10.1186/s40621-015-0044-5 (PMC5005715; doi:10.1186/s40621-015-0044-5)
Supplement: Additional file 1: — Injury syndrome inclusion/exclusion criteria. [file 40621_2015_44_MOESM1_ESM.docx]

**Additional file 1: Injury syndrome inclusion/exclusion criteria:**

**Traffic-related injury to pedal cyclist**

Includes any: “BIKE” or “BICYCLE”

Excludes any: “MOTORCYC” or [misspellings of motorcycle] or “CYCLIC”

**Traffic-related injury to pedestrian**

Includes any: “PED“ or “PEDES” or “STRUCK BY CAR” or “HIT BY CAR”
Excludes any: “BIKE” or “BICYCLE” or [misspellings of bicycle] or “MOTORCYC” or [misspellings of motorcycle]

**Traffic-related injury to motor vehicle occupant**

Includes any: “MVA” or [variations of spacing/periods on M.V.A.] or “MVC”

Conditional inclusions: (“CAR” or [misspellings of car] or “VAN” or “TRUCK” or “SUV” or “AUTO” or

“TAXI” or “MOTOR” or “TRAFFIC” or “VEHICLE” or [misspellings of vehicle] or “MOTORCYC” or

[misspellings of motorcycle] or “BUS ) and (“STRUCK” or “ACC” or “CRASH” or “HIT”)

Excludes any: “BIKE” or “BICYCLE” or [misspellings of bicycle] or “CYCL” or “PED” or “PEDES” or

“STRUCK BY” or “HIT BY” or “FALL” or “FELL” or “SLIPPED”

**Fall-related injury**

Includes any: “FALL” or “FELL” or “SLIP” or “TRIP” or “STUMBLE”

Conditional inclusions, among patients age <1 year or >64 years: “CONCUS” or “TBI” or “BRAIN INJ” or “HEAD INJ” or “HEAD TRA” or “HEAD TRUA”

Excludes any: “FELLING” or “STRAIN” or “INTRACT” or “NON” or “MVA” or “MVC”

**Firearm-related injury**

Includes any: “GSW” or “GUNSHOT” or “GUN SHOT” or “WAS SHOT” or “GOT SHOT” or “SHOT IN” or “BEEN SHOT” or “SHOT WITH” or “SHOT AT”

Conditional inclusions: “GUN” and “WOUND”

Excludes any: “BB” or [variations and alternate spellings of B.B.] or “INFECT” or “REACTION” or “BLOODSHOT” or “BLOOD SHOT” or “LAZER” or “TAZER” or “LASER” or “TASER” or “PAINT BALL” or “STUN” or “PELLE” or [misspellings of PELLET] or “NAIL” or “PENICIL” or “TETANUS” or [misspelling of tetanus] or “FLU˽” or “INSULIN” or “RABIES” or “PNEUMONIA” or “GSWEL” or “GSWOL” or “FOLLOW-UP” or “FOLLOW UP” or [variations of FU] or “OLD GUN” or “OLD GSW” or “GET A SHOT” or “YEAR AGO” or “YR AGO”

**Assault-related stab injury**

Includes any: “STAB” or [tense variations of stab] or “SLASH”

Condition inclusions: (“CUT” or “BLADE” or “KNIFE” or “LAC”) and ([variations and misspellings of ASSAULT] or “FIGHT” or “ROBBED” or “WAS JUMPED”)

Excludes any: “ROLLERBLADE” or “PRIOR” or “STABLE” or [misspellings of stable] or “UNSTAB” or “ESTAB” or “POSTABOR” or “EPIPEN” or “EPI PEN” or “SELF” or “PEN” or “PENCIL” or “THREAT” or “ACCID” or “INFECT” or “RADIAT” or “CHEST” or “PAIN” or “STABISMUS” or “SUICI” or “DEPRES” or “IDEATION” or “SLASH WRIST” or “OLD WOUND”

Conditional exclusion, 1: “STABBIN” or [misspellings of stabbing] and (“PAIN” or “FEELING” or “PN” or “PX” or “CRAMP” or “HEADACHE” or [misspellings of headache]

Conditional exclusions, 2: “STAB” and [tense variations of try] and (“FIGHT” or “ROB” are not present)

**SAS code**

/*TRAFFIC-RELATED INJURY TO PEDAL CYCLIST*/

bike=**0**;

IF (prxmatch("m/BIKE|BICYLE|BICL|CYCL/",cc))

AND (prxmatch("m/MOTO[RC ][CY ][YC]|CYCLIC/",cc)=**0**)

THEN bike=**1**;

/*TRAFFIC-RELATED INJURY TO PEDESTRIAN*/

ped=**0**;

IF prxmatch("m/\bPED |PEDES|STRUCK BY CAR|HIT BY CAR/",cc)

AND (prxmatch("m/BIKE|BICYCLE|BIC[YL]|CYCL|MOTO[RC ][CY ][YC]/",cc)=**0**)

THEN ped=**1**;

/*TRAFFIC-RELATED INJURY TO MOTOR VEHICLE OCCUPANT*/

mv=**0**;

IF (prxmatch("m/M[ .]V[ .]A|MVA|MVC/",cc)

OR (prxmatch("m/\bC[ A]R|CAR|VAN|\bTRUCK|SUV|AUTO|CAB|TAXI|MOTOR|TRAFFIC|VE[ICH][IHC][ILCH][LCE]|MOTO[RC ][CY ][YC]|BUS/",cc))

AND (prxmatch("m/STRUCK|ACC|CRASH|\bHIT/",cc)))

AND (prxmatch("m/BIKE|BICYLE|BICL|CYCL|\bPED |PEDES|STRUCK BY|HIT BY|FALL|FELL|SLIPPED/",cc)=**0**)

THEN mv=**1**;

/*FALL-RELATED INJURY*/

fallnohi=**0**;

IF prxmatch("m/F[EA]LL|SLIP|TRIP|STUMBLE/",cc)

AND (prxmatch ("m/FELLING/",cc)=**0**)

THEN fallnohi=**1**;

headinj=**0**;

IF (prxmatch("m/CONCUS|TBI |BRAIN INJ|HEAD INJ/",cc)

OR (prxmatch ("m/HEAD/",cc) AND prxmatch("m/TRA|TRUA/",cc))

AND (prxmatch("m/STRAIN|INTRACT|NON|MVA|MVC/",cc)=**0**))

THEN headinj=**1**;

fall=**0**;

/*Head injuries included for infants and older adults because >80% of traumatic brain injuries are caused by falls in those age groups*/

IF fallnohi=**1** THEN fall=**1**;

else IF agenum=**0** and headinj=**1** THEN fall=**1**;

else IF agenum ge **65** and headinj=**1** THEN fall=**1**;

/*FIREARM-RELATED INJURY*/

gun=**0**;

IF ((prxmatch("m/\bGUN|\bGON /",cc) AND prxmatch("m/WOUND/",cc))

OR prxmatch("m/\bGSW|GUNSHOT|GUN SHOT|WAS SHOT|GOT SHOT|SHOT IN|BEEN SHOT|SHOT WITH|SHOT AT/",cc))

AND (prxmatch("m/BEE[B ][BE]|B\.B\.|\bBB|B B |BIBI|BEBE|INFECT|REACTION|BLOODSHOT|BLOOD SHOT|[LT]A[SZ]ER|PAINT BALL

|\bSTUN |PEL[EL]|NAIL|PENICIL|TEATNUS|TETANUS|\bFLU |INSULIN|RABIES|PNEUMONIA|GSWEL|GWSOL|FOLLOW[ -]UP|F\/U|OLD GUN

|OLD GSW|GET A SHOT/",cc)=**0**)

AND (prxmatch("m/YEAR|YR/",cc)=**0** AND prxmatch("m/AGO/",cc)=**0**)

THEN gun=**1**;

/*ASSAULT-RELATED STABBING INJURY*/

stab=**0**;

IF (prxmatch("m/STAB|STABB[IE]|SLASH/",cc)

OR (prxmatch("m/CUT|BLADE|KNIFE|LAC/",cc) AND prxmatch("m/ASSAUL|ASSULT|FIGHT|ROBBED|WAS JUMPED/",cc)))

THEN stab=**1**;

IF (prxmatch("m/ROLLERBLADE|PRIOR|STAB[AI]L|STABLE|STABLIZ|UNSTAB|ESTAB|POSTABOR|EPIPEN|EPI PEN|SELF|\bPEN |PENCIL|THREAT|ACCID|INFECT|RADIAT|CHEST PAIN|STABISMUS|SUICI|DEPRES|IDEATION/",cc)

OR (prxmatch("m/STABBIN|STABIN/",cc) AND prxmatch("m/PAIN|FEELING|PN |PX |CRAMP|HEADAC[HE][HE]/", cc))

OR (prxmatch("m/SLASH/",cc) AND prxmatch("m/WRIST/",cc))

OR (prxmatch("m/STAB/",cc) AND prxmatch("m/\bTRY|\bTRIED|\bTRYING/",cc) AND prxmatch("m/FIGHT|ROB/",cc)=**0**)

OR (prxmatch("m/OLD/",cc) AND prxmatch("m/WOUND/",cc)))

THEN stab=**0**;
