# Supplementary material for: Utility of a near real-time emergency department syndromic surveillance system to track injuries in New York City
Source: Inj Epidemiol. 2015 Jun 1;2(1):11. doi: 10.1186/s40621-015-0044-5 (PMC5005715; doi:10.1186/s40621-015-0044-5)
Supplement: Additional file 2: — ICD-9-CM E-codes for injury types. [file 40621_2015_44_MOESM2_ESM.docx]

**Additional file 2: ICD-9-CM E-codes for injury types:**

*Traffic-related injury to pedal cyclist:*

E810.6, E811.6, E812.6, E813.6, E814.6, E815.6, E816.6, E817.6, E818.6, E819.6, E800.3, E801.3, E802.3, E803.3, E804.3, E805.3, E807.3, E820.6, E821.6, E822.6, E823.6, E824.6, E825.6, E826.1, E826.9, E827.1, E828.1, E829.1

*Traffic-related injury to pedestrian:*

E810.7, E811.7, E812.7, E813.7, E814.7, E815.7, E816.7, E818.7, E819.7

*Traffic-related injury to motor vehicle occupant:*

E810.0, E810.1, E811.0, E811.1, E812.0, E812.1, E813.0, E813.1, E814.0, E814.1, E815.0, E815.1, E816.0, E816.1, E817.0, E817.1, E818.0, E818.1, E819.0, E819.1

*Fall-related injury:*

E880, E881, E882, E883, E884, E885, E886, E888, E957, E968.1, E987

*Firearm-related injury:*

E922.0, E922.1, E922.2, E922.3, E922.8, E922.9, E955.0, E955.1, E955.2, E955.3, E955.4, E965.0, E965.1, E965.2, E965.3, E965.4, E970, E985.0, E985.1, E985.2, E985.3, E985.4, E979.4

*Assault-related stabbing injury:*

E966
