# Supplementary material for: Utility of a near real-time emergency department syndromic surveillance system to track injuries in New York City
Source: Inj Epidemiol. 2015 Jun 1;2(1):11. doi: 10.1186/s40621-015-0044-5 (PMC5005715; doi:10.1186/s40621-015-0044-5)
Supplement: Additional file 3: — Distribution of injury-related ED visits by quarter of year, ED SS vs. SPARCS, NYC, 2008-2010. [file 40621_2015_44_MOESM3_ESM.docx]

**Additional file 3: Distribution of injury-related ED visits by quarter of year, ED SS vs. SPARCS, NYC, 2008-2010**

|  |  |  | ***3-year totals, 2008-2010*** | |  |
| --- | --- | --- | --- | --- | --- |
| **Injury Type** | **Quarter of Year** | | **ED SS Proportion** | **SPARCS Proportion** | **Absolute % Point Difference** |
| Traffic-related injury to pedal cyclist | January-March | | 9.3 | 8.4 | 0.9 |
|  | April-June | | 32.8 | 33.2 | 0.4 |
|  | July-September | | 42.4 | 43.0 | 0.6 |
|  | October-December | | 15.5 | 15.5 | 0.0 |
| Traffic-related injury to pedestrian | January-March | | 22.0 | 23.8 | 1.8 |
|  | April-June | | 26.2 | 25.1 | 1.1 |
|  | July-September | | 24.7 | 23.7 | 1.0 |
|  | October-December | | 27.1 | 27.4 | 0.3 |
| Traffic-related injury to motor vehicle occupant | January-March | | 21.4 | 21.9 | 0.5 |
|  | April-June | | 27.0 | 26.4 | 0.6 |
|  | July-September | | 27.1 | 26.5 | 0.6 |
|  | October-December | | 24.5 | 25.2 | 0.7 |
| Fall-related injury | January-March | | 23.5 | 23.7 | 0.2 |
|  | April-June | | 25.8 | 25.7 | 0.1 |
|  | July-September | | 26.1 | 25.8 | 0.3 |
|  | October-December | | 24.6 | 24.8 | 0.2 |
| Firearm-related injury | January-March | | 17.1 | 17.1 | 0.0 |
|  | April-June | | 28.6 | 27.7 | 0.9 |
|  | July-September | | 32.5 | 32.3 | 0.2 |
|  | October-December | | 21.8 | 22.9 | 1.1 |
| Assault-related stabbing injury | January-March | | 21.0 | 21.0 | 0.0 |
|  | April-June | | 27.4 | 27.3 | 0.1 |
|  | July-September | | 29.1 | 29.1 | 0.0 |
|  | October-December | | 22.5 | 22.6 | 0.1 |

Notes: ^a^ Column percentages may not add up to 100% due to rounding and/or missing data
