# Supplementary material for: Utility of a near real-time emergency department syndromic surveillance system to track injuries in New York City
Source: Inj Epidemiol. 2015 Jun 1;2(1):11. doi: 10.1186/s40621-015-0044-5 (PMC5005715; doi:10.1186/s40621-015-0044-5)
Supplement: Additional file 4: — Distribution of injury-related ED visits by neighborhood of residence, ED SS vs. SPARCS, NYC, 2008-2010. [file 40621_2015_44_MOESM4_ESM.docx]

**Additional file 4: Distribution of injury-related ED visits by neighborhood of residence, ED SS vs. SPARCS, NYC, 2008-2010**

|  | **Traffic-related injury to pedal cyclist^a^** | | | **Traffic-related injury to pedestrian^a^** | | | **Traffic-related injury to motor vehicle occupant^a^** | | |
| --- | --- | --- | --- | --- | --- | --- | --- | --- | --- |
| **Neighborhood of residence** | **ED SS Proportion** | **SPARCS Proportion** | **Absolute % Point Difference** | **ED SS Proportion** | **SPARCS Proportion** | **Absolute % Point Difference** | **ED SS Proportion** | **SPARCS Proportion** | **Absolute % Point Difference** |
| Kingsbridge-Riverdale | 0.7 | 0.7 | 0.0 | 0.5 | 0.8 | 0.3 | 0.6 | 0.6 | 0.0 |
| Northeast Bronx | 1.4 | 1.6 | 0.2 | 2.1 | 2.2 | 0.1 | 3.1 | 3.1 | 0.0 |
| Fordham-Bronx Park | 2.9 | 2.7 | 0.2 | 2.2 | 3.8 | 1.6 | 2.9 | 3.1 | 0.2 |
| Pelham-Throgs Neck | 3.3 | 2.8 | 0.5 | 4.0 | 3.0 | 1.0 | 4.4 | 3.2 | 1.2 |
| Crotona-Tremont | 2.3 | 2.4 | 0.1 | 1.4 | 3.6 | 2.2 | 2.3 | 2.5 | 0.2 |
| High Bridge-Morrisania | 3.3 | 2.5 | 0.8 | 1.8 | 3.0 | 1.2 | 3.0 | 2.3 | 0.7 |
| Hunts Point-Mott Haven | 1.7 | 1.8 | 0.1 | 1.6 | 1.8 | 0.2 | 1.9 | 1.3 | 0.6 |
| Greenpoint | 2.7 | 2.6 | 0.1 | 1.3 | 1.2 | 0.1 | 0.6 | 0.5 | 0.1 |
| Downtown-Heights-Slope | 2.9 | 4.5 | 1.6 | 1.6 | 2.1 | 0.5 | 1.3 | 1.5 | 0.2 |
| Bedford Stuyvesant-Crown Heights | 4.5 | 4.8 | 0.3 | 4.5 | 5.3 | 0.8 | 4.9 | 4.6 | 0.3 |
| East New York | 3.2 | 2.8 | 0.4 | 2.9 | 3.1 | 0.2 | 3.7 | 3.5 | 0.2 |
| Sunset Park | 0.8 | 2.6 | 1.8 | 1.8 | 2.2 | 0.4 | 0.9 | 1.1 | 0.2 |
| Borough Park | 1.6 | 3.7 | 2.1 | 4.2 | 3.4 | 0.8 | 2.8 | 2.3 | 0.5 |
| East Flatbush-Flatbush | 3.4 | 3.6 | 0.2 | 4.4 | 4.7 | 0.3 | 5.5 | 5.4 | 0.1 |
| Canarsie-Flatlands | 1.5 | 1.8 | 0.3 | 2.2 | 2.7 | 0.5 | 4.5 | 4.3 | 0.2 |
| Bensonhurst-Bay Ridge | 0.8 | 2.2 | 1.4 | 1.7 | 2.2 | 0.5 | 1.5 | 1.7 | 0.2 |
| Coney Island-Sheepshead Bay | 2.4 | 3.4 | 1.0 | 3.5 | 3.3 | 0.2 | 3.6 | 2.5 | 1.1 |
| Williamsburg-Bushwick | 6.2 | 5.2 | 1.0 | 3.2 | 3.3 | 0.1 | 2.5 | 2.3 | 0.2 |
| Washington Heights-Inwood | 5.2 | 2.9 | 2.3 | 2.7 | 2.8 | 0.1 | 2.2 | 1.8 | 0.4 |
| Central Harlem-Morningside Heights | 4.0 | 1.9 | 2.1 | 2.5 | 2.0 | 0.5 | 1.7 | 1.1 | 0.6 |
| East Harlem | 2.1 | 1.6 | 0.5 | 1.6 | 1.6 | 0.0 | 1.0 | 0.8 | 0.2 |
| Upper West Side | 3.9 | 2.0 | 1.9 | 2.0 | 1.2 | 0.8 | 0.7 | 0.5 | 0.2 |
| Upper East Side | 2.0 | 2.2 | 0.2 | 1.5 | 1.8 | 0.3 | 0.5 | 0.6 | 0.1 |
| Chelsea-Clinton | 3.4 | 1.8 | 1.6 | 1.6 | 1.4 | 0.2 | 0.5 | 0.4 | 0.1 |
| Gramercy Park-Murray Hill | 1.7 | 1.4 | 0.3 | 1.6 | 1.5 | 0.1 | 0.4 | 0.4 | 0.0 |
| Greenwich Village-Soho | 2.2 | 1.3 | 0.9 | 0.6 | 0.6 | 0.0 | 0.2 | 0.2 | 0.0 |
| Union Square-Lower East Side | 4.0 | 3.3 | 0.7 | 2.2 | 1.8 | 0.4 | 0.8 | 0.7 | 0.1 |
| Lower Manhattan | 0.9 | 0.6 | 0.3 | 0.4 | 0.4 | 0.0 | 0.3 | 0.4 | 0.1 |
| Long Island City-Astoria | 1.4 | 2.5 | 1.1 | 3.5 | 2.4 | 1.1 | 1.8 | 1.7 | 0.1 |
| West Queens | 3.3 | 6.2 | 2.9 | 9.1 | 5.2 | 3.9 | 4.8 | 4.4 | 0.4 |
| Flushing-Clearview | 0.9 | 1.5 | 0.6 | 2.8 | 3.1 | 0.3 | 2.0 | 2.5 | 0.5 |
| Bayside-Little Neck | 0.4 | 0.4 | 0.0 | 0.5 | 0.5 | 0.0 | 0.6 | 0.7 | 0.1 |
| Ridgewood-Forest Hills | 1.3 | 1.8 | 0.5 | 2.4 | 2.0 | 0.4 | 1.8 | 1.7 | 0.1 |
| Fresh Meadows | 0.5 | 0.6 | 0.1 | 0.8 | 0.9 | 0.1 | 0.9 | 1.1 | 0.2 |
| Southwest Queens | 3.8 | 3.1 | 0.7 | 3.1 | 3.3 | 0.2 | 4.0 | 3.7 | 0.3 |
| Jamaica | 2.9 | 2.7 | 0.2 | 2.8 | 3.3 | 0.5 | 5.2 | 4.9 | 0.3 |
| Southeast Queens | 1.5 | 1.4 | 0.1 | 1.8 | 1.6 | 0.2 | 4.0 | 3.3 | 0.7 |
| Rockaway | 0.4 | 0.8 | 0.4 | 0.6 | 0.8 | 0.2 | 1.4 | 1.3 | 0.1 |
| Port Richmond | 0.6 | 1.0 | 0.4 | 1.4 | 1.1 | 0.3 | 1.1 | 1.8 | 0.7 |
| Stapleton-St. George | 0.7 | 1.0 | 0.3 | 1.3 | 1.4 | 0.1 | 1.3 | 2.9 | 1.6 |
| Willowbrook | 0.2 | 0.4 | 0.2 | 0.6 | 0.7 | 0.1 | 0.7 | 1.8 | 1.1 |
| South Beach-Tottenville | 0.3 | 1.1 | 0.8 | 0.7 | 1.3 | 0.6 | 0.8 | 4.8 | 4.0 |

|  | **Fall-related injury^a^** | | | **Firearm-related injury^a^** | | | **Assault-related stabbing injury^a^** | | |
| --- | --- | --- | --- | --- | --- | --- | --- | --- | --- |
| **Neighborhood of residence** | **ED SS Proportion** | **SPARCS Proportion** | **Absolute % Point Difference** | **ED SS Proportion** | **SPARCS Proportion** | **Absolute % Point Difference** | **ED SS Proportion** | **SPARCS Proportion** | **Absolute % Point Difference** |
| Kingsbridge-Riverdale | 1.3 | 1.1 | 0.2 | 0.4 | 0.4 | 0.0 | 0.5 | 0.5 | 0.0 |
| Northeast Bronx | 2.3 | 2.6 | 0.3 | 3.3 | 3.0 | 0.3 | 2.7 | 3.4 | 0.7 |
| Fordham-Bronx Park | 3.6 | 4.2 | 0.6 | 3.0 | 4.0 | 1.0 | 3.8 | 5.9 | 2.1 |
| Pelham-Throgs Neck | 4.0 | 3.8 | 0.2 | 4.5 | 3.9 | 0.6 | 4.9 | 4.9 | 0.0 |
| Crotona-Tremont | 2.9 | 3.5 | 0.6 | 2.8 | 6.3 | 3.5 | 3.7 | 6.6 | 2.9 |
| High Bridge-Morrisania | 3.6 | 3.5 | 0.1 | 4.0 | 6.1 | 2.1 | 6.6 | 6.6 | 0.0 |
| Hunts Point-Mott Haven | 1.9 | 2.3 | 0.4 | 2.7 | 3.7 | 1.0 | 4.8 | 4.5 | 0.3 |
| Greenpoint | 1.1 | 1.1 | 0.0 | 0.4 | 0.3 | 0.1 | 0.9 | 0.7 | 0.2 |
| Downtown-Heights-Slope | 1.7 | 2.9 | 1.2 | 2.0 | 2.0 | 0.0 | 1.2 | 1.3 | 0.1 |
| Bedford Stuyvesant-Crown Heights | 3.6 | 3.6 | 0.0 | 12.9 | 11.9 | 1.0 | 6.4 | 6.9 | 0.5 |
| East New York | 2.8 | 2.4 | 0.4 | 7.6 | 6.9 | 0.7 | 4.4 | 4.2 | 0.2 |
| Sunset Park | 0.9 | 1.6 | 0.7 | 0.7 | 0.6 | 0.1 | 1.4 | 1.9 | 0.5 |
| Borough Park | 3.2 | 3.8 | 0.6 | 0.6 | 0.7 | 0.1 | 1.3 | 1.5 | 0.2 |
| East Flatbush-Flatbush | 3.4 | 3.2 | 0.2 | 7.2 | 7.4 | 0.2 | 4.0 | 5.0 | 1.0 |
| Canarsie-Flatlands | 2.4 | 2.1 | 0.3 | 2.9 | 3.0 | 0.1 | 1.9 | 2.0 | 0.1 |
| Bensonhurst-Bay Ridge | 1.5 | 2.2 | 0.7 | 0.4 | 0.5 | 0.1 | 0.9 | 1.1 | 0.2 |
| Coney Island-Sheepshead Bay | 4.3 | 3.6 | 0.7 | 1.9 | 1.6 | 0.3 | 2.5 | 2.0 | 0.5 |
| Williamsburg-Bushwick | 3.5 | 3.1 | 0.4 | 5.4 | 4.6 | 0.8 | 4.9 | 4.5 | 0.4 |
| Washington Heights-Inwood | 3.7 | 3.1 | 0.6 | 2.1 | 2.0 | 0.1 | 4.8 | 3.6 | 1.2 |
| Central Harlem-Morningside Heights | 3.1 | 2.1 | 1.0 | 4.0 | 3.9 | 0.1 | 3.7 | 3.2 | 0.5 |
| East Harlem | 2.0 | 1.9 | 0.1 | 2.8 | 2.4 | 0.4 | 2.5 | 2.1 | 0.4 |
| Upper West Side | 2.8 | 2.0 | 0.8 | 0.7 | 0.5 | 0.2 | 1.0 | 0.7 | 0.3 |
| Upper East Side | 2.0 | 2.2 | 0.2 | 0.2 | 0.2 | 0.0 | 0.4 | 0.3 | 0.1 |
| Chelsea-Clinton | 2.0 | 1.4 | 0.6 | 0.4 | 0.3 | 0.1 | 1.3 | 0.7 | 0.6 |
| Gramercy Park-Murray Hill | 1.4 | 1.2 | 0.2 | 0.5 | 0.3 | 0.2 | 0.9 | 0.5 | 0.4 |
| Greenwich Village-Soho | 0.9 | 0.7 | 0.2 | 0.1 | 0.1 | 0.0 | 0.4 | 0.2 | 0.2 |
| Union Square-Lower East Side | 2.4 | 1.8 | 0.6 | 1.0 | 0.9 | 0.1 | 1.3 | 1.1 | 0.2 |
| Lower Manhattan | 0.5 | 0.5 | 0.0 | 0.3 | 0.3 | 0.0 | 0.3 | 0.2 | 0.1 |
| Long Island City-Astoria | 2.5 | 2.1 | 0.4 | 1.0 | 0.8 | 0.2 | 1.4 | 1.0 | 0.4 |
| West Queens | 4.7 | 4.5 | 0.2 | 1.9 | 2.1 | 0.2 | 4.6 | 4.3 | 0.3 |
| Flushing-Clearview | 2.3 | 2.4 | 0.1 | 0.3 | 0.4 | 0.1 | 1.3 | 0.8 | 0.5 |
| Bayside-Little Neck | 0.4 | 0.5 | 0.1 | 0.0 | 0.0 | 0.0 | 0.1 | 0.1 | 0.0 |
| Ridgewood-Forest Hills | 1.9 | 1.9 | 0.0 | 0.5 | 0.5 | 0.0 | 1.2 | 1.2 | 0.0 |
| Fresh Meadows | 0.9 | 1.0 | 0.1 | 0.2 | 0.2 | 0.0 | 0.3 | 0.3 | 0.0 |
| Southwest Queens | 3.4 | 2.7 | 0.7 | 1.8 | 1.5 | 0.3 | 2.5 | 2.3 | 0.2 |
| Jamaica | 3.2 | 2.9 | 0.3 | 4.7 | 4.5 | 0.2 | 3.3 | 3.2 | 0.1 |
| Southeast Queens | 1.8 | 1.5 | 0.3 | 1.5 | 1.7 | 0.2 | 1.4 | 1.3 | 0.1 |
| Rockaway | 0.6 | 0.7 | 0.1 | 2.7 | 2.4 | 0.3 | 1.1 | 1.4 | 0.3 |
| Port Richmond | 0.8 | 1.1 | 0.3 | 1.1 | 1.0 | 0.1 | 1.0 | 1.6 | 0.6 |
| Stapleton-St. George | 1.0 | 1.9 | 0.9 | 1.3 | 1.5 | 0.2 | 1.1 | 1.6 | 0.5 |
| Willowbrook | 0.5 | 1.1 | 0.6 | 0.2 | 0.3 | 0.1 | 0.2 | 0.3 | 0.1 |
| South Beach-Tottenville | 0.5 | 2.6 | 2.1 | 0.2 | 0.2 | 0.0 | 0.4 | 0.5 | 0.1 |

Notes: ^a^ Column percentages may not add up to 100% due to rounding and/or missing data
